# The class II histone deacetylases as therapeutic targets for Parkinson’s disease

**DOI:** 10.1042/NS20200001

**Published:** 2020-06-09

**Authors:** Martina Mazzocchi, Louise M Collins, Aideen M. Sullivan, Gerard W. O'Keeffe

**Affiliations:** 1 Department of Anatomy and Neuroscience, and Cork Neuroscience Centre, Western Gateway Building, University College Cork, Cork, Ireland; 2 Department of Physiology, Western Gateway Building, University College Cork, Cork, Ireland; 3 APC Microbiome Institute, University College Cork, Cork, Ireland

**Keywords:** Parkinsons disease, α-synuclein, neuroprotection, HDAC, dopamine neuron, histone deacetylase

## Abstract

Parkinson’s disease (PD) is a progressive neurodegenerative disorder characterised by specific motor impairments. The neuropathological hallmarks of PD include progressive degeneration of midbrain dopaminergic neurons, and loss of their axonal projections to the striatum. Additionally, there is progressive accumulation and spread of intracellular aggregates of α-synuclein. Although dopamine-replacement pharmacotherapy can treat PD symptoms in the short-term, there is a critical need for the development of disease-modifying therapies based on an understanding of the underlying disease mechanisms. One such mechanism is histone acetylation, which is a common epigenetic modification that alters gene transcription. A number of studies have described alterations in histone acetylation in the brains of PD patients. Moreover, α-synuclein accumulation has been linked to alterations in histone acetylation and pharmacological strategies aimed at modulating histone acetylation are under investigation as novel approaches to disease modification in PD. Currently, such strategies are focused predominantly on pan-inhibition of histone deacetylase (HDAC) enzymes. Inhibition of specific individual HDAC enzymes is a more targeted strategy that may allow for future clinical translation. However, the most appropriate class of HDACs that should be targeted for neuroprotection in PD is still unclear. Recent work has shed new light on the role of class-II HDACs in dopaminergic degeneration. For this reason, here we describe the regulation of histone acetylation, outline the evidence for alterations in histone acetylation in the PD brain, and focus on the roles of class II HDACs and the potential of class-II HDAC inhibition as a therapeutic approach for neuroprotection in PD.

## Introduction

Parkinson’s disease (PD) is the second most common neurodegenerative disorder affecting approximately 0.2% of the global population and 1% of people aged over 60 years of age [1,2]. It is estimated that the prevalence of neurodegenerative diseases such as PD will triple by 2050. However, despite 50 years of research, the etiology of PD remains largely unknown and there is currently no disease-modifying therapy [3,4]. PD is characterised by both motor and non-motor symptoms; the cardinal motor symptoms include resting tremor, rigidity, akinesia and postural instability [5,6]. Autonomic dysfunctions are found in 70–80% of patients, with the majority of impairments relating to the parasympathetic system. Such symptoms include orthostatic or postural hypotension, constipation, dysphagia and urinary symptoms [6]. Disease progression is due to gradual degeneration of dopaminergic neurons in the midbrain substantia nigra pars compacta (SNpc), resulting in a deficit of dopamine neurotransmission in the striatum [7]. The key pathology involves the presence of abnormal, intracellular aggregates of the presynaptic protein, α-synuclein; these aggregates are termed Lewy bodies [8,9]. The misfolding of oligomeric α-synuclein into these aggregates is thought to trigger the neuroinflammation and neurodegeneration that is characteristic of PD [10,11]. The majority of PD cases are idiopathic, with only 5–10% of cases caused by inheritable genetic mutations. There is much evidence linking idiopathic PD to environmental factors, for example, exposure to pesticides such as rotenone and paraquat [12]. However, no unifying mechanism has yet emerged to explain the aetiology of idiopathic PD, and ageing remains the main risk factor [13]. Recently, there has been a growing interest in epigenetic dysregulation and the roles that it may play in the etiology and progression of PD (for reviews see [14,15]).

## Histone acetylation and histone deacetylation

**Figure 1:**
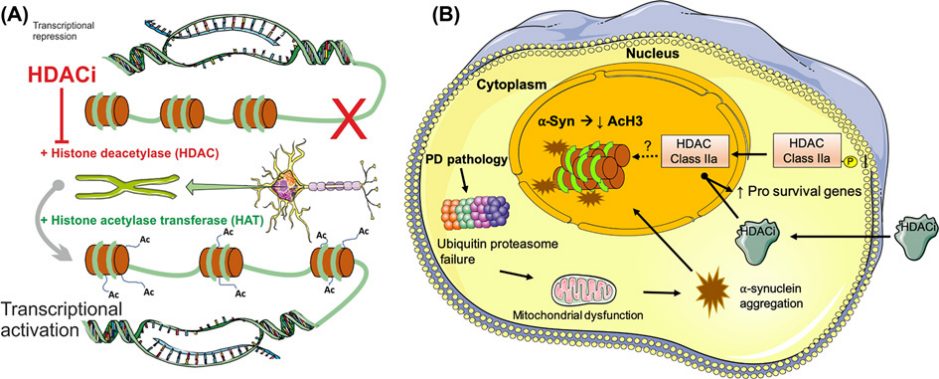
Class II HDAC inhibitors as potential neuroprotective agents for PD therapy (**A**) Histone acetylation is a common epigenetic mechanism and is mediated by histone acetyltransferases (HAT) enzymes, which acetylate (Ac) lysine residues in the N-terminal of histone proteins. This neutralises the positive charge on the histone tail, decreasing DNA–histone interactions, which renders the DNA more accessible for transcription factor binding and thus enhances gene expression (transcriptional activation). Histone deacetylation is the opposite process and is mediated histone deacetylases (HDACs) enzymes, which remove acetyl groups, which renders the DNA less accessible for transcription factor binding, thus inhibiting gene expression (transcriptional repression). HDAC inhibitors (HDACi) are a novel class of drugs that inhibit the activity of HDACs, increasing acetylation of lysine residues on histone proteins, thus inhibiting transcriptional repression (indicated by the red X) and enhancing transcriptional activation. (**B**) In PD, intracellular events, including failure of the ubiquitin proteasome system and mitochondrial dysfunction, culminate in the accumulation of α-synuclein aggregates in neurons. This results in the impairment of histone acetylation and the translocation of Class IIa HDACs from the cytoplasm to the nucleus. Treatment with inhibitors of Class IIa HDACs (HDACi) can reverse these effects and increase levels of pro-survival genes, supporting a role for Class IIa HDACi as neuroprotective therapies for PD.

Epigenetic modifications involve physical changes to DNA or to histone proteins, resulting in alterations in gene expression. Histone acetylation and deacetylation are epigenetic modifications that play key roles in many fundamental cellular processes, such as transcriptional regulation and chromatin remodeling. These reactions are catalysed by enzymes which possess histone acetyltransferase (HAT) or histone deacetylase (HDAC) activity. Acetylation involves the addition of an acetyl functional group, whereas deacetylation is the reverse reaction, which is the removal of an acetyl group [16]. Acetylation of histone proteins results in a loose chromatin state which allows for transcriptional activation, whereas histone deacetylation results in a tight compact chromatin structure which suppresses transcriptional activity [17] (Figure 1A). The process of histone deacetylation is regulated by the HDACs, an enzyme superfamily which includes several subtypes: class I, class IIa, class IIb, class III and class IV [18]. Class I (HDAC1, HDAC2, HDAC3, HDAC8) and class IIa (HDAC4, HDAC5, HDAC7, HDAC9) HDACs have structural homology and both classes are composed of Zn^2+^-dependent enzymes [19,20] (Table 1). HDAC4, HDAC5, HDAC7 and MITR (a splice variant of HDAC9) shuttle between the nucleus and the cytoplasm due to their nuclear localisation sequence, while full-length HDAC9 is located only in the nucleus [21,22]. HDAC4 and HDAC5 undergo activity-dependent export from the nucleus which is modulated by calcium ion influx through L-type calcium channels and synaptic NMDA receptors [23]. The class IIb HDAC10 is structurally similar to class I and IIa, in that it contains a single Zn^2+^-dependent catalytic domain, while the class IIb HDAC6 has an unconventional structure, containing two independent Zn^2+^-dependent catalytic domains. Class IV (HDAC11) and class III (also referred to as sirtuins, of which several isoforms exist) HDACs are functionally and structurally different from the other HDACs [24,25]. The balance between HDAC and HAT activities is tightly controlled in healthy cells, but there is evidence that disruption of this balance may play roles in the pathogenesis of several diseases, including PD.

**Table 1:** Structural organisation and intracellular localisation of individual members of the HDAC classes

HDAC class	HDAC name	Domain organisation	Cellular localisation
Class I	HDAC1	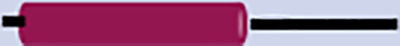	Nucleus/Axon
Class I	HDAC2	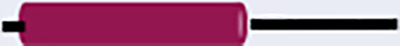	Nucleus
Class I	HDAC3	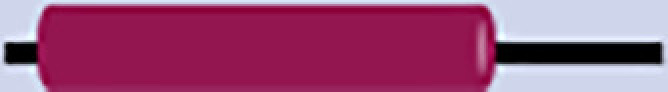	Nucleus/Axon
Class I	HDAC8	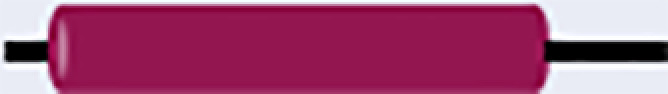	Nucleus/Cytoplasm
Class IIa	HDAC4	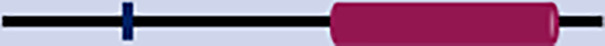	Nucleus/Axon
Class IIa	HDAC5	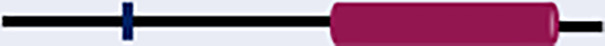	Nucleus/Axon
Class IIa	HDAC7	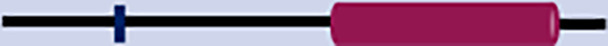	Nucleus/Cytoplasm
Class IIa	HDAC9	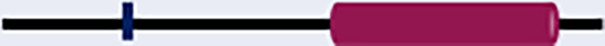	Nucleus/Cytoplasm
Class IIb	HDAC6	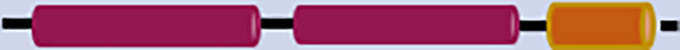	Nucleus/Axon
Class IIb	HDAC10	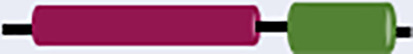	Nucleus/Cytoplasm
Class III	SIRTS	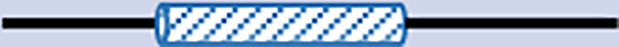	Nucleus/Cytoplasm/Mitochondria
Class IV	HDAC11	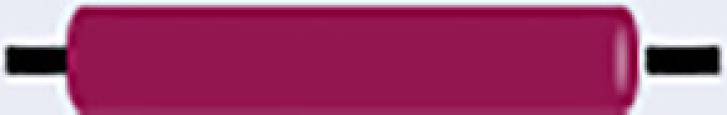	Nucleus

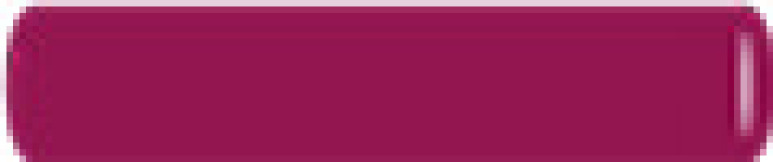
 Deacetylase domain, 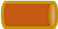 ZnF-UBP domain, 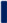 MEF binding domain, 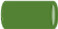 Leucine rich-domain, 
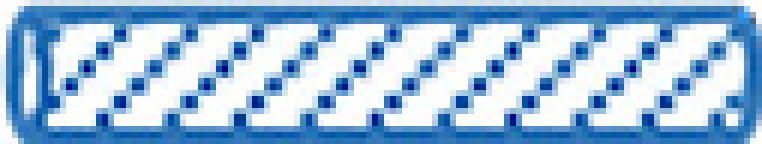
 SIRT deacetylase domain

## Alterations in histone acetylation in Parkinson’s disease

Although there is mounting evidence for dysregulation of the epigenome in PD [14,15,26], to date only a few studies have examined the levels of histone acetylation in the PD brain. An important study by Park et al. examined histone acetylation in SNpc post mortem samples from five PD patients (mean age = 73.8 ± 4.4 years) and compared this to five age-matched control samples (74.4 ± 3.5 years) [27]. Western blotting revealed a marked increase in acetylation of a commonly acetylated histone residue, Lysine 9 (AcH3-K9), in addition to increases in AcH2A-K5, AcH2B-K15, and AcH4-K5 in three of the five PD patient samples, compared with the controls [27]. The increases in AcH2A-K5, AcH3-K9 and AcH4-K5 were not seen in the cerebellar cortex from the PD patients, indicating specificity of these changes to the SNpc [27]. In addition, the authors used immunostaining to show high levels of AcH3 in 83% of dopaminergic (tyrosine hydroxylase (TH)-immunopositive) midbrain neurons in the PD SNpc, which was not found in non-dopaminergic cells in these samples, and was only seen in 2% of dopaminergic neurons in the SNpc from controls. In a complementary study, Harrison et al. examined histone acetylation, as well as HDAC levels, in SNpc samples from a gender-balanced cohort composed of eight patients with early (Braak stage 3 /4) PD (79.3 ± 1.8 years), twelve late (Braak stage 6) PD cases (79.3 ± 1.8 years) and ten age-matched controls (82.1 ± 1.9 years) [28]. They found significant increases in the levels of AcH3-K9 (normalised to β-actin expression) in protein extracts from SNpc of late-stage PD cases compared with controls. Moreover, the increases in AcH3-K9 levels significantly correlated with disease progression [28], as measured by Braak staging [29]. While these two studies found increases in acetylated Lysine 9 in the PD SN, another study reported increases in AcH3-K18 and AcH3-K23, and decreases in AcH3-K9 levels, in samples of primary motor cortex from PD patients, without increases in global levels of Histone 3 [30]. Despite slight differences between studies, there is a general trend towards reports of increased histone acetylation in the PD brain, and in particular in the SN.

Two studies have examined the expression in PD brain samples of HDACs, the enzymes that regulate histone acetylation. One study found decreases in protein expression of HDAC1, HDAC2, HDAC6 and SIRT1 in PD midbrain samples compared with controls [27]. In contrast, Harrison and colleagues reported that there were no significant differences in the expression of class I HDACs (HDAC1, 2, 3 and HDAC8), class IIa HDACs (HDAC4, 5, 7 and HDAC9), class IIb HDACs (HDAC6, 10) or class III (SIRT 1, 2) in PD SNpc, compared with age-matched controls [28]. A transcriptome study on post mortem SN tissue from eight late-stage PD (Braak stage 5/6) patients (78.5 ± 1.9 years), compared with eight age-matched, non-demented controls (75.5 ± 7.6 years) reported a 1.6-fold higher level of HDAC6 expression, and a 1.65-fold higher level of HAT1 expression in the PD samples [31]. This is consistent with the increases in AcH3 levels in the PD SN reported in the studies described above [27,28].

However, there is not absolute agreement between studies in terms of the effects of PD pathology on HDAC and HAT expression and thus further research will be important. Moreover, it is possible that any changes in histone acetylation levels in the PD brain may not only result from changes in HDAC or HAT expression, but be caused by alterations in the activities of HDACs or HATs. To our knowledge, this is currently unknown and is an important avenue for future research. Future studies on characterisation of the levels and activities of individual HDACs in the Parkinsonian brain will be crucial in order to justify the exploration of HDAC inhibitors as therapeutics.

There have been some advances in the study of individual classes of HDACs in laboratory models of PD, as well potential neuroprotective effects of their inhibition. For the purposes of this review, we focus on studies that have explored the biology of class II HDACs in models of PD.

## Class IIa HDACs as therapeutic targets for PD

The class IIa HDACs are HDAC4, HDAC5, HDAC7 and HDAC9 [32]. Recent work has implicated HDAC4 as a neurotoxic mediator in models of PD [33]. Specifically, treatment of cultured midbrain neurons from A53T α-synuclein transgenic mice with the dopaminergic neurotoxin, 1-methyl-4-phenylpyridinium ion (MPP+), led to nuclear accumulation of HDAC4. This was not seen in cultures prepared from MPP+-treated wild-type control mice, or in untreated cultures from A53T α-synuclein transgenic mice. Nuclear accumulation of HDAC4 was also detected in the SN of A53T α-synuclein transgenic mice following administration of 1-methyl-4-phenyl-1,2,3,6-tetrahydropyridine (MPTP; the precursor of MPP+) [33]. The class IIa HDACs are unique in that they shuttle between the cytoplasm and nucleus, a process which is mediated by phosphorylation at serine residues [34]. Wu and colleagues used truncated forms of HDAC4 which were exclusively localised to the cytoplasm or the nucleus to show that nuclear-localised HDAC4 exerts neurotoxic effects, whereas cytoplasmic HDAC4 does not [33]. These studies show that HDAC4 can contribute to the vulnerability of mutant α-synuclein-expressing midbrain dopaminergic neurons, suggesting that class IIa HDAC inhibitors may have neuroprotective effects.

In support of this, neuroprotective effects of class IIa HDAC inhibition *in vitro* have been shown by our group [35]. In light of the fact that the early stages of PD are characterised by rapid axonal degeneration [36,37], we set out to examine the effects of the class IIa HDAC inhibitor, MC1568 [38], on neurite growth and neuroprotection in three cell models [35]. These models were SH-SY5Y cells (a widely-used model of human dopaminergic neurons [39]), cultured rat ventral midbrain dopaminergic neurons and cultured sympathetic neurons from the superior cervical ganglion. The latter were chosen due to substantial evidence for sympathetic denervation occurring in PD [40,41]. Pharmacological inhibition of class IIa HDACs using MC1568 promoted neurite growth and branching, and led to a significant increase in AcH3-S11K15 levels, in all three cell types. Importantly, MC1568 treatment also promoted cell survival and protected against the neurotoxic effects of MPP+ in each of these cell models [35].

**Figure 2:**
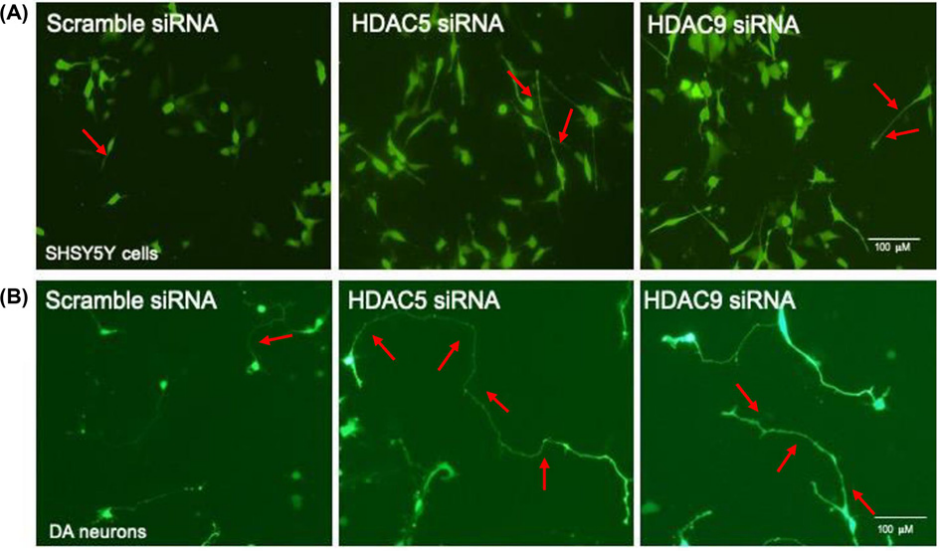
HDAC5 and HDAC9 are negative regulators of neurite length *in vitro* Representative photomicrographs of (**A**) SH-SY5Y cells and (**B**) primary cultures of embryonic day 14 rat ventral mesencephalon, at (**A**) 72 h or (**B**) 24 h after co-transfection with GFP-tagged protein and with control scrambled siRNA or siRNA targeting HDAC5 or HDAC9, as indicated. Neurites are indicated by red arrows; scale bar = 100 μm. Data are modified from Mazzocchi et al. [42].

Building on this work, our recent study examined whether any class IIa HDACs were co-expressed with markers of dopaminergic neurons in the human SN [42]. This analysis was based on the evidence that correlated patterns of gene expression can reflect functional association [43]. Of all of the class IIa HDACs, only the expression of *HDAC5* and *HDAC9* had a positive correlation with expression of three markers of dopaminergic neurons (*TH, GIRK2* and *ALDH1A1*) in the human SN [42]. This was confirmed in the mouse SN, where it was found that transcripts for *Hdac5* and *Hdac9* were present, and that HDAC5 and HDAC9 co-localised with TH-positive dopaminergic neurons [42]. Additionally, short interfering RNA (siRNA)-mediated inhibition of HDAC5 or HDAC9 (Figure 2A,B), or pharmacological inhibition of these using the class IIa HDAC inhibitor, MC1568, promoted neurite growth in models of dopaminergic neurons by upregulating the activity of the BMP-Smad pathway [42]. Furthermore, siRNAs targeting either HDAC5 or HDAC9 promoted neurite growth in cells overexpressing wild-type or A53T-α-synuclein, and treatment with MC1568 protected cultured rat dopaminergic neurons against the neurotoxin, MPP^+^ [42].

In support of the above evidence for neurotoxic effects of class IIa HDACs, other investigators studying neuroblastoma cells have reported that siRNA-mediated inhibition of HDAC5 results in significant increases in the expression of transcripts for markers of dopaminergic neuronal differentiation (including *th, gap-43*, and β*-III tubulin*), as well as promoting neurite outgrowth [44]. Moreover, injury-induced nuclear export of HDAC5 has been shown to be required for axonal regeneration in peripheral sensory neurons, since localisation of HDAC5 exclusively to the nucleus prevented axonal regeneration [45]. Similarly, another study reported that nuclear export of HDAC9 is required for axonal branching in thalamocortical neurons [46]. Specifically, this study showed that nuclear export of HDAC9 occurs during the process of thalamocortical axonal branching, and that overexpression of a form of HDAC9 that is retained in the nucleus resulted in inhibition of this branching [46]. In contrast with the above studies that showed detrimental effects of class II HDACs on neurons, HDAC7 expression has been found to protect cerebellar granule neurons from apoptosis through a deacetylase-independent mechanism [47]. However, it is important to note that HDAC7 is expressed at much lower levels than HDAC4, 5 and 9 in the SN *in vivo* [33].

Collectively, these studies are largely consistent and point to a detrimental effect of nuclear localisation of class IIa HDACs. Furthermore, they suggest that cytoplasmic shuttling of class IIa HDACs may be essential for the survival and maintenance of nigrostriatal dopaminergic neurons. This is supported by the neuroprotective effects of class IIa HDAC inhibition [35,42], which rationalises the further study of class IIa HDAC inhibition in neuroprotective therapy for PD, and in particular the investigation of their effects in models of α-synucleinopathy.

## Class IIb HDACs as therapeutic targets for PD

The class IIb HDACs are HDAC6 and HDAC10 [48–50]. While midbrain dopaminergic neurons have been shown to express HDAC6 and HDAC10 [51], to date most studies on class IIb HDACs have focused on HDAC6 and little is known about the function of HDAC10 in these neurons. Interest in HDAC6 in the context of PD arose with the demonstration that this HDAC plays a crucial role in the clearance of misfolded and aggregated proteins [52,53]. Specifically, knockdown of HDAC6 resulted in failure of cellular clearance of misfolded protein aggregates from the cytoplasm, lack of formation of aggregsomes (key organelles for the clearance of toxic misfolded protein aggregates), and hypersensitivity to the accumulation of misfolded proteins, resulting in cell death [52]. Moreover, HDAC6 has been found to be strongly expressed in α-synuclein- and ubiquitin-positive Lewy bodies in brain sections from patients with PD [52]. A functional role of HDAC6 in midbrain dopaminergic neurons was demonstrated by a study showing that HDAC6 knockout in *Drosophila* exacerbated α-synuclein-induced degeneration of dopaminergic neurons and resulted in motor dysfunction [54]. Moreover, overexpression of HDAC6 could protect against the detrimental effects of α-synuclein on dopaminergic neurons. In that study, HDAC6 was shown to physically interact with oligomeric α-synuclein and to play a crucial role in the formation of Lewy body-like inclusions [54]. Subsequently, that group used the ubiquitin-proteasome system (UPS) impairment mouse model of PD, induced by injection of lactacystin to the medial forebrain bundle, to further study the role of HDAC6 [55]. They found that although HDAC6 protein was expressed at lower levels in the SN than in hippocampus, cortex or striatum in the adult mouse brain, lactacystin injection led to a selective increase in HDAC6 expression in dopaminergic neurons in the SN [55]. In these cells, HDAC6 was located in the cytoplasm within perinuclear inclusion bodies that were structurally similar to aggregsomes [55]. Moreover, intraperitoneal injection of the pan-HDAC inhibitor, trichostatin A (which inhibits all Class I, II and IV HDACs), exacerbated lactacystin-induced nigrostriatal dopaminergic neuron degeneration and associated behavioural deficits, and increased α-synuclein oligomer levels in the SN [55]. These data are consistent with previous reports showing the involvement of HDAC6 in the clearance of misfolded proteins [52,54]. Moreover, these effects of HDAC6 appear to require its deacetylase activity, since treatment with the selective HDAC6 inhibitor, tubacin, exacerbated lactacystin-induced cell death in primary cultures of rat ventral midbrain, whereas treatment with niltubacin, an analog of tubacin that does not inhibit HDAC6 deacetylase activity, did not [55]. These studies are important as they show that the deacetylase activity of HDAC6 is required for its protective effects on dopaminergic neurons. Collectively, the studies described above suggest that HDAC6 plays a protective role in midbrain dopaminergic neurons. However, while the above findings are convincing, a number of recent reports have raised the possibility that the role of HDAC6 in nigral dopaminergic neurons may depend on the cellular context. Mutations in *leucine-rich repeat kinase 2* (*LRRK2*) are the most common genetic cause of PD and association studies have shown that the *LRRK2* locus is a risk factor for sporadic PD [56–60]. Expression of the pathogenic LRRK2 mutants, R1441C and Y1669C, but not of LRRK2-G2019S or of LRRK2 itself, disrupted axonal transport *in vitro* in rat cortical neurons and *in vivo* in *Drosophila* motor neurons, as well as altering motor behaviour in *Drosophila* [61]. Intriguingly, knockdown of HDAC6 was shown to effectively restore axonal transport and motor behaviour in *Drosophila* that expressed the LRRK2 mutants (R1441C and Y1669C). Moreover, systemic administration of trichostatin A was sufficient to restore axonal transport and improve these behavioural deficits, even when administered after the onset of the behavioural symptoms [61]. In agreement with these findings, the HDAC6 inhibitor, tubastatin A, has been reported to rescue both MPP+-induced reductions in dopaminergic neurons and MPP+-induced metabolic impairments, in studies on *Zebrafish* [62]. However, in contrast with the study by Godena et al., administration of tubastatin A did not rescue impairments in spontaneous movements or in sensorimotor reflexes induced in *Zebrafish* by MPP+, whereas a pan-HDAC inhibitor, 4-phenylbutyrate, improved both of these motor symptoms [62]. Additional evidence for a neuroprotective effect of HDAC6 inhibition came from a study on a mouse model of PD, involving intrastriatal administration of the dopaminergic neurotoxin 6-hydroxydopamine (6-OHDA) [63]. At one week after 6-OHDA injection, there was a significant increase in transcript and protein levels of HDAC6 in dopaminergic neurons in the SN [63]. Pharmacological inhibition of HDAC6 using tubastatin A improved apomorphine-induced asymmetrical rotations and prevented 6-OHDA-induced decreases in TH expression in the SN; however, this study did not report the numbers of nigral dopaminergic neurons or the degree of striatal innervation [63]. Collectively, these studies show that HDAC6 inhibition can have neuroprotective effects, depending on the cellular context. In future studies, it will be important to determine how α-synuclein affects HDAC6 expression and whether HDAC6 inhibition protects against or exacerbates α-synuclein-induced neurotoxicity *in vivo.*

## Effects of α-synuclein on histone acetylation

Accumulation of α-synuclein into aggregates, the pathological hallmark of PD, is thought to result in disruption of the balance of HATs and HDACs, resulting in downstream alterations in gene expression. There have been many studies on the regulation of SNCA, the gene that encodes synuclein, by epigenetic mechanisms, particularly DNA methylation (reviewed by 64,65). However, the effects that accumulation and aggregation of α-synuclein may have on histone acetylation are not clear. There is some evidence for a role of histone acetylation in the neurotoxic effects of α-synuclein accumulation in cultured cells. For example, the toxic effects of α-synuclein overexpression in SH-SY5Y cells have been found to be dependent on the association of α-synuclein with histones in the nucleus, resulting in decreased histone H3 acetylation [66]. Furthermore, these toxic effects could be rescued by treatment of the SH-SY5Y cells with histone deacetylase inhibitors. That study also reported similar effects in transgenic *Drosophila*, in which α-synuclein was found to bind directly to histones and to reduce histone H3 acetylation [66]. Nuclear localisation of α-synuclein in *in vivo* PD models has been previously reported by another group [67]. Another *in vitro* study found that α-synuclein reduces the levels of p300 and its HAT activity in cultured N27 dopaminergic neuronal cells, and that p300 expression is reduced in nigral dopaminergic neurons in α-synuclein-transgenic mice [68]. In PD, intracellular events, including failure of the ubiquitin proteasome system and mitochondrial dysfunction, culminate in the accumulation of a-synuclein aggregates in neurons. The observation that nuclear α-synuclein can affect histone acetylation supports a need for future studies to investigate the effects of HDAC inhibition on the intracellular pathways involved in dopaminergic neuronal survival and axonal maintenance in models of PD, with the ultimate aim of testing the potential of Class II HDAC inhibitors as neuroprotective therapies for PD (Figure 1B).

## Conclusions and future perspectives

There is mounting evidence for neuroprotective effects of HDACs inhibitors on midbrain dopamine neurons. Most of this has come from studies on cultured cells and animal models of PD. In particular, there is strong rationale for exploring the potential neuroprotective effects of Class IIa HDACs inhibitors in animal models of PD, in order to advance the translation of these studies towards clinical application. A number of studies have reported neuroprotective effects of the pan-HDAC inhibitor, valproic acid, on the nigrostriatal pathway in *in vivo* animal models of PD, such as rotenone-treated rats [67], MPTP-treated mice [69], lactacystin-treated rats [70] and LRRK2 mutant mice [71]. However, to date very few *in vivo* studies have applied inhibitors of specific classes of HDACs. There is some evidence for neuroprotective potential of class III inhibitors in *in vivo* PD models. For example, Outeiro and colleagues identified an inhibitor of SIRT2, a class III HDAC, which showed neuroprotective effects in an α-synuclein-overexpressing *Drosophila* model of PD [72]. Another study reported protective effects of a brain-penetrant SIRT2 inhibitor in the MPTP mouse model of PD [73]. However, the neuroprotective potential of class II inhibitors is under question due to a recent study showing that the selective class III HDAC inhibitor, nicotinamide, exacerbated the neurotoxic effects of intranigral injection of the irreversible proteasome inhibitor, lactacystin, in adult rats [74].

This highlights the need for further work to elucidate the selective actions of specific HDAC classes on midbrain dopaminergic neurons, to ensure that appropriate candidates are advanced in preclinical studies. Additionally, further studies are needed to clearly elucidate the acetylation levels of individual HDACs in the brains of PD patients, particularly in nigrostriatal dopaminergic neurons, but also in other neuronal pathways that are known to be affected by this disease. In order for HDAC inhibitors to have therapeutic efficacy, it will be critical to ascertain that endogenous levels of the specifically-targeted HDACs are maintained within the dopaminergic neurons of the PD midbrain.

## Abbreviations

6-OHDA: 6-hydroxydopamine
AcH2A-K5: acetylated histone 2 A lysine 5
AcH2B-K15: acetylated histone 2 B lysine 15
AcH3-K19: acetylated histone 3 lysine 19
AcH3-K23: acetylated histone 3 lysine 23
AcH3-K9: acetylated histone 3 lysine 9
AcH3-S11K15: acetylated histone 3 serine 11 lysine 15
AcH4-K5: acetylates histone 4 lysine 5
HAT: histone acetylase
HDAC: histone deacetylase
MPP+: 1-methyl-4-phenylpyridinium
MPTP: 1-methyl-4-phenyl-1,2,3,6-tetrahydropyridine
PD: Parkinson’s disease
SiRNA: short interfering RNA
SN (pc): substantia nigra (pars compacta)
TH: tyrosine hydroxylase
UPS: ubiquitin/proteasome system
